# The Demography, Longevity and Mortality of Bullmastiffs Attending Veterinary Practices in Australia

**DOI:** 10.3390/ani14233419

**Published:** 2024-11-26

**Authors:** Abigail Carney, Peter Williamson, Rosanne M. Taylor

**Affiliations:** Sydney School of Veterinary Science, University of Sydney, Camperdown, NSW 2006, Australia; acar8976@uni.sydney.edu.au

**Keywords:** Bullmastiff, demography, longevity, mortality, VetCompass, electronic patient record, epidemiology

## Abstract

The Bullmastiff is a giant breed of dog, and there is evidence the breed is predisposed to cancer and musculoskeletal disorders, though the disease investigation of the breed is limited. This study reports on the demography, longevity and mortality of Bullmastiffs attending veterinary practices in Australia over a ten-year period. VetCompass Australia collects patient data from veterinary practices across Australia for epidemiological analysis. All patient records of Bullmastiffs available in the VetCompass Australia database during this decade period were reviewed, with demographic information on the breed inclusive of coat colour, sex, neuter status, weight and location collated. Standardised veterinary diagnostic (VeNom) codes for the most appropriate cause of death were assigned to deceased dogs. The population comprised 2771 Bullmastiffs with an overall median age of 2.8 years. Within the group, 1259 were female (45.4%), 1491 were male (53.8%), and 21 dogs (0.8%) had no recorded sex or neuter status. Dogs grew rapidly in their first year, with an average gain of approximately 1 kg every 10 days. A slower growth rate continued in their second year, and growth plateaued as adulthood was reached, with the mean body weight of adult male dogs (46.6 kg) being heavier than that of females (40.5 kg). The age at death for the group was 8.5 years. The most common causes of death in the breed were mass lesions (28.2%), old age (9.9%), musculoskeletal-related disease (9.9%) and neurological (5.3%) and behavioural disorders (4.8%). Neutering was protective against mortality from urogenital causes (OR: 0.14; CI: 0.02–0.52; *p* = 0.003) and had a positive effect on longevity. This study provides demographic and health information on a population of Bullmastiffs attending veterinary practices in Australia, which will benefit evidence-based veterinary decisions for this breed. Additionally, the results may assist owners and breeders in making informed decisions on health risks and breeding programmes in the population.

## Simple Summary

The disease investigation of companion animals is important for the clinical management of disease as well as the control of disease risk factors. The Bullmastiff is a giant brachycephalic dog breed which has been reported to have an increased risk of lymphoma, musculoskeletal and ocular disorders, as well as a shortened lifespan, although an in-depth disease investigation of the Bullmastiff has not been conducted. This study aimed to provide a comprehensive retrospective study of the breed, investigating the demography, longevity and mortality of Bullmastiffs attending veterinary practices in Australia over a ten-year period from 2008 to 2017. This was achieved through the systematic review of patient records from veterinary practices participating in the VetCompass research collaboration. This study described the demography, body weight, longevity and mortality of the Bullmastiff, revealing the study population to have a median age of 2.8 years and a mean body weight of 46.6 kg for males and 40.5 kg for females. The dogs had a median longevity of 8.5 years, and the major causes of mortality were mass lesions, old age and musculoskeletal, neurological and behavioural disorders. Desexing was found to significantly reduce the risk of mortality due to urogenital causes. The results of this study may assist in the veterinary care, health management, breeding guidelines and welfare of Bullmastiffs.

## 1. Introduction

Bullmastiffs are a large breed of dog originally bred to perform as guard-dogs in 19th-century England. The breed is the result of a mixture of 60% Mastiff to 40% Bulldog, resulting in a breed with gigantic stature (standard weight range of 41–50 kg for adult females; 50–59 kg for adult males) and brachycephalic features. The Australian National Kennel Club (ANKC) describes this utility breed as having both “powerful physical and mental attributes”, but as with many large breeds, there has been a decline in Bullmastiff registration in Australia in recent years.

Reduced longevity has been associated with large and brachycephalic breeds and with breed development [1,2,3,4]. A negative correlation between body size and lifespan has been reported for a number of breeds in UK studies, for example, the Great Dane (6.5 years) and Irish Wolfhound (7.0 years), when compared to the median longevity of other pure-breed dogs (11 years 3 months) [5]. Similarly, studies based on veterinary records in the UK have reported a decreased lifespan (7.2 years) of the Bulldog [3]. Overall, the exploration of veterinary data in the UK revealed that the Dogue de Bordeaux, a giant brachycephalic breed, had the shortest lifespan (5.5 years) of the reported UK breeds [6]. In keeping with these trends of brachycephalic and giant dog breeds, owner surveys in the UK also showed a decreased longevity (8.6 and 7.5 years, respectively) for the Bullmastiff breed [5,7].

An increased disease risk in brachycephalic and giant breeds has also been reported with the potential for impact on mortality. For example, a UK owner survey revealed that cancer-related deaths were common for Bullmastiffs (34.2%) and Giant Schnauzers (41.0%). Likewise, giant breeds insured in Sweden had high tumour-related mortality, and Bernese Mountain Dogs in Denmark commonly died or were euthanised due to cancer (34.2%) [7,8,9]. Furthermore, accelerated ageing and growth in large breeds increases the risk of appendicular osteoarthritis and other degenerative disorders (e.g., elbow dysplasia and osteochondritis dissecans) [10,11]. The Bulldog has a high disease burden, commonly presenting with cutaneous (28.6%), ophthalmological (18.0%), aural (13.0%), enteropathy (11.6%) and upper respiratory tract issues, with heart disease (11.8%), neoplasia (10.9%) and brain disorders (9.1%) being the most common causes of morbidity and mortality in a UK patient data study of Bulldogs [3]. Additionally, an analysis of UK owner survey data revealed that neoplasia (21.8%) was the most common cause of euthanasia in the Dogue de Bordeaux [12]. Patient data from Australia correspond with insurance data from Bullmastiff patients indicating that the breed has an increased risk of lymphoma (OR: 6.76 and 4.8, respectively) [13,14]. Moreover, the analysis of veterinary records in the USA has revealed an increased risk of musculoskeletal disorders (OR: 3.16 osteoarthritis; OR: 2.80 ruptured cranial cruciate ligament) in Bullmastiffs. The breed has also been linked to a genetic mutation (T4R) which puts the breed at risk of degenerative retinal disorders (progressive retinal atrophy) [15]. Despite these findings, the impact of these disorders on death, longevity and euthanasia decisions in this breed has yet to be examined.

VetCompass Australia follows a similar design to the programme established in the UK. It is a research collaboration between seven veterinary schools and two hundred veterinary practices across Australia, extracting clinical data in the form of electronic patient records (EPRs) to examine companion animal populations [16]. These records provide histories of the health of patients which are stored in an online database. Large datasets, including EPR, insurance data and owner surveys, have enhanced the knowledge of the common health problems in dog breeds, and the analysis of these data provides potential opportunities for early detection, management and selective breeding to reduce these conditions [17]. VetCompass UK studies have investigated mortality, disease and longevity in a range of breeds [18], but the Bullmastiff breed has not been a focus.

This study reports the demography, mortality and longevity of Bullmastiff dogs that visited veterinary practices participating in the VetCompass Australia programme over a ten-year period. We hypothesized that the primary signalment factors, neuter status, weight and coat colour, contribute to longevity and mortality. The results of this study provide evidence to inform improvements in breed health programmes, dog welfare, health management and veterinary diagnoses related to the breed [19].

## 2. Materials and Methods

### 2.1. Population Demographics

**Figure 1:**
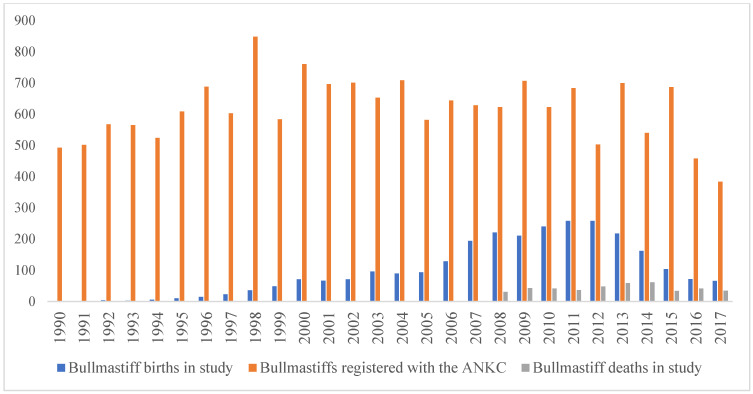
Number of births and deaths of Bullmastiffs attending veterinary care at practices participating in the VetCompass Australia programme from 1 January 2008 to 31 December 2017 compared to the number of Bullmastiffs registered with the ANKC for the corresponding years.

The study population included all Bullmastiffs in the period of 1 January 2008–31 December 2017 which attended any of the 200 veterinary practices participating in the VetCompass Australia programme [16]. All deidentified records were selected from the total VetCompass database with breed specification recorded by veterinarians. The dogs within this study visited a veterinary practice in this ten-year time frame and had at least one EPR. Dogs attending practices outside of this period were not included in this study, and one dog was excluded due to an incomplete record. Each record in VetCompass had an associated unique animal identifier with demographic information on species, breed, date of birth, date of death (if available), sex, neuter status, body weight and post code as well as examination text [20]. Neuter status was defined by the final available EPR neuter value and was combined with sex: female entire, female neutered, male entire and male neutered. All EPR data were extracted into Excel (Microsoft Office Excel 2013, Microsoft Corp, Seattle, WA, USA), followed by data cleaning and analysis. Any records where dogs did not meet the breed phenotype/colours were considered misidentified and excluded. Demographic information on coat colour, sex, neuter status, weight, location and median age of population was collated (Table 1). The annual birth and death rates of dogs in this study were compared to the number of Bullmastiffs registered with the ANKC from 1990 to 2017 (Figure 1).

### 2.2. Body Weight

**Figure 2:**
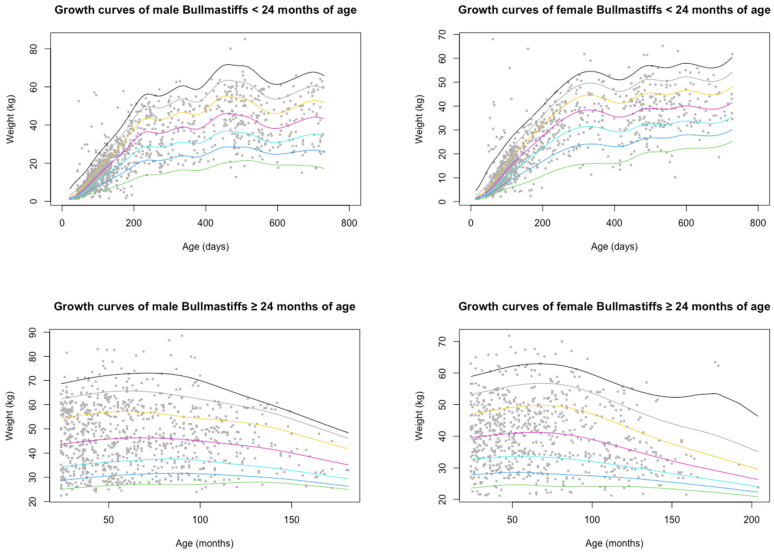
Body weights and age for “well” male and female Bullmastiffs attending veterinary practices participating in the VetCompass Australia programme from 1 January 2008 to 31 December 2017 (males n = 970; females n = 777). Coloured lines indicate growth centiles.

**Figure 3:**
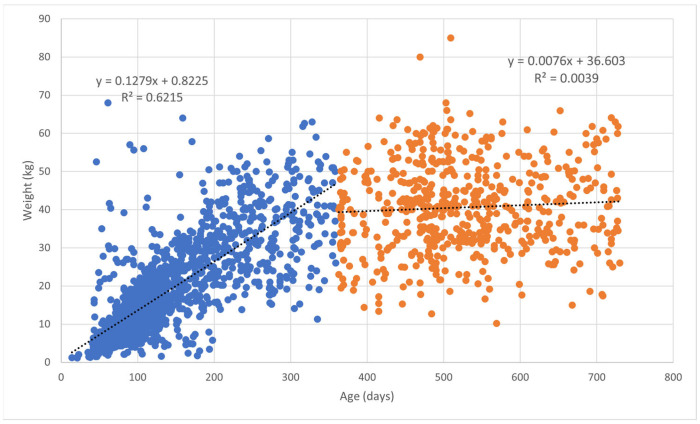
Body weights of young Bullmastiffs (<24 months) attending veterinary care at practices participating in the VetCompass Australia programme from 1 January 2008 to 31 December 2017. The blue data points represent Bullmastiffs from 0 to 12 months, whilst the orange data points represent Bullmastiffs from 12 to 24 months.

Body weight data were obtained from dogs visiting veterinary practices for wellness/routine visits, as to include only healthy Bullmastiffs in the body weight population. This was inclusive of visits for desexing, vaccinations, microchipping and routine visits where the animal was examined and confirmed to be in good health. EPRs were excluded if the veterinarian reported that the dog was overweight or underweight. The data were separated into young dogs (<24 months of age) and adult dogs (≥24 months), with age determined by date of birth and age at consultation. Outliers (≤6 weeks of age and ≤1 kg in weight) in the young dog (<24 months of age) population were excluded. Adult dogs (≥24 months) which were less than half of the lightest ANKC breed standard weight for bitches (20.5 kg) and more than double the heaviest breed standard weight for dogs (118 kg) were excluded for the purpose of weight analyses. The body weight curves were produced by the method of Tambalis et al. [21] using the Lambda Mu and Sigma (LMS) approach (Figure 2). The LMS function implemented in R was used to construct growth curve centiles, and the centile function plotted the centile curves. The centiles displayed were the 3rd, 10th, 25th, 50th, 75th, 90th and 97th centiles. The mean body weights of the adult weight records (male neutered, male entire, female neutered, female entire) were analysed including the body weight of the deceased population using the patient’s last available body weight prior to death (Table 1). The body weight data were grouped into weight intervals (Table 1). Adult records that were at least 10% under (37 kg for females; 45 kg for males) or over (55 kg for females; 65 kg for males) the ANKC weight range were reported as under- or overweight for body weight data compared to the breed standard (Table 1 and Table 2). Regression analysis of the body weight over time for the first and second years of life was used to define the growth rates of the breed (Figure 3).

### 2.3. Longevity and Mortality

Following the exclusion of 5 records, a subset of 433 deceased dog records were available for analysis. The records were excluded because they were incomplete or inaccurate (i.e., conflicting sex or neuter status listed for a dog, multiple dogs listed under one unique patient identifier) or inappropriate for this study (i.e., incorrect breed, records outside of the study period).

The veterinarian’s examination texts from EPRs were used to assign VeNom definitive diagnostic codes for the cause of euthanasia or death for each dog. The VeNom code list is a standardised list of clinical veterinary terms and diagnoses used for fine- and group-level diagnosis. The cause of death was extracted from the EPR when recorded at the time of death or following death. Where the cause was not evident, additional veterinary interpretation of the clinical information provided identified the cause of death or where this was unclear or missing it was recorded as unknown. When a fine-level diagnosis was determined, the researcher documented whether the dog had been euthanised, had died without assistance or the mode of death was not specified (Table 2). Dogs were allocated to cause-of-death categories: accident, traumatic injury, advanced age, unspecified, problematic behaviour and disorder, and health condition. “Old age” was designated as a cause-of-death category when the clinical record described old age as the cause of death, no underlying condition was reported, the age of the dog was greater than the upper quartile for the population (11.2 years), and a diagnosis could not be extracted from the clinical notes. One VeNom code was used to identify the main cause of death recorded by the veterinarian; secondary and third VeNom codes were given when other conditions contributed to death but were not the sole cause. The fine-precision-level diagnoses were then categorised into diagnosis groups; for example, canine parvovirus was grouped into an infectious diagnosis (Table 3) [3,18,22]. Relevant information on the conditions in which dogs were kept (i.e., nutrition, exercise, housing, reproductive status) was assessed when relevant to the cause of death.

Statistical analysis using Chi-squared tests for categorical data and Student’s *t*-tests for continuous data were conducted using RStudio or functions in Excel (Microsoft Corp) with significance set at *p* < 0.05. Confidence intervals for prevalence were calculated using the asymptotic and Wilson approximation (for disorder groups with less than ten dogs) methods in RStudio. Signalment factors (neuter status, age, body weight and colour) were analysed against the grouped-precision-level disorders using a generalised linear model (GLM) to determine whether they had a significant effect on death. Univariate and then multivariate logistic regression (to account for confounding factors) was performed. Survival analysis based on sex, weight, colour, neuter status and the longevity of grouped causes of death was included. Kaplan–Meir curves and Cox proportional hazard models were used for univariate and multivariate analysis, producing a log rank *p* value and hazard ratios (Table 2) [23].

## 3. Results

### 3.1. Demography of Population

There was a total population of 2771 Bullmastiffs attending the 200 participating veterinary practices from 2008 to 2017. The majority of dogs were located within the state of New South Wales (NSW) (70.4%) with the remainder in Victoria (VIC) and Queensland (QLD) (Table 1). These states represent approximately 78% of the total Australian population. There was a significant difference in the proportion of female and male dogs: 45.4% were female, 53.8% were male (*p* < 0.001), and 0.8% of dogs had an unrecorded sex or neuter status. Of the sub-population with sex and neuter status recorded, 48.4% of females were neutered compared to 38.6% of males. Red (45.2%) was the most frequent coat colour followed by fawn (20.2%); the brindle (16.0%), other (10.9%) and not reported (7.7%) coat colour categories were less common. The median age of Bullmastiffs in the population was 2.8 years (IQR 0.8–6.3, range 0.0–25.3), with the most frequent age group being <3.0 (51.4%) (Table 1). There was a decline in the number of births of dogs in this study between 2010–2011 and 2015–2017 (Figure 1). The annual births as a percent of Bullmastiffs visiting veterinarians per year show similar trends, with 45.4% of patients under 1 year of age in 2011 but only 17.2% in 2017. ANKC registration (Figure 1) showed a similar decline, with a reduction in Bullmastiff registrations of 38.4% in 2017 when compared to 2008.

### 3.2. Body Weight

Body weight is a key indicator of health status, and certain conditions may be related to an accelerated growth rate. At a breed level, body size has been linked to lifespan in dogs. To establish normal growth rates and weight variation in Bullmastiffs, body weight data were extracted from wellness and routine visits, which included 1747 dogs representing 63% of all recovered records. There were 777 female dogs (44.5%), 315 of which were adults (40.5%), and 970 males (55.5%), 387 of which were adults (39.9%). There was a significant difference in the proportion of males to females in the body weight data (*p* < 0.001). The body weights of dogs participating in wellness visits were normally distributed with the most frequent weight range being 30.0–34.0 kg for adult females (17.0%) and 55.0–59.0 kg for adult males (14.9%) (Table 1). As expected, the mean (±SD) body weight of the adult males was significantly heavier (46.6 ± 12.6 kg) than that of the adult females (40.5 ± 10.4 kg; *p* < 0.001). The growth of Bullmastiffs under 24 months old was most rapid in the first 200–400 days (Figure 2), reaching on average approximately 1 kg every 10 days for the first 300 days. Proportionally, weight gain in the first year was significantly greater than that in the second year (*p* < 0.001), when growth began to plateau (Figure 3). The body weight curves of the adults mostly remained at a plateau until they decreased from 100 to 150 months (8.3–12.5 years) (Figure 2). The mean body weight (35.5 ± 6.7 kg) of older males (≥12.5 years) was significantly less than the mean body weight (46.9 ± 12.6 kg) of younger adult males (<12.5 years; *p* < 0.001). This was also the case for females, with the mean body weight (33.8 ± kg) of older females (≥12.5 years) being significantly less than the mean body weight (40.7 ± 10.3) of younger adult females (<12.5 years; *p* = 0.01). These data are unusual for healthy dogs at this stage of life.

### 3.3. Demography of Deceased Population

Bullmastiffs that died during the study period from 2008 to 2017 comprised 15.6% of the total study population, with the majority in NSW (70.9%) (Table 1). Amongst the deceased, the proportion of females was significantly lower than that of males (*p* = 0.007): 187 females (43.2%), 243 males (56.1%) and 3 (0.7%) with unrecorded sex. Females were more likely to be neutered (67.9%) than entire (*p* < 0.001), whilst the proportion of entire to neutered males (51.0%) was similar. The most common coat colour among the deceased dogs was red (47.3%), followed by fawn (18.5%), brindle (17.8%), not described (8.5%) and other (7.9%). Within the deceased group, the mean adult body weight for males was significantly heavier (44.3 ± 15.5 kg) than that of the female dogs (39.5 ± 14.0 kg; *p* = 0.006). The data represented 73.2% of the total deceased group, with 92.4% of the data being adult Bullmastiffs (≥2.0 years).

### 3.4. Longevity

There has been considerable interest in dogs as a model system for understanding lifespan and longevity in recent years. In the Bullmastiff dogs studied here, the overall distribution of longevity was bimodal, with peaks in the <1 and 8–10 years categories and a median longevity of 8.5 years (IQR 5.3–11.2; range 0.0–18.6). When the sex and neuter status of deceased dogs was analysed, the longevity of male dogs (8.5 years; IQR 5.2–11.0; range 0.0–18.0) did not differ significantly from that of female dogs (8.3 years; IQR 5.4–11.5; range 0.0–18.6) (*p* = 0.38) (Table 2). However, the median longevity of neutered dogs (9.0 years; IQR 6.0–11.6; range 0.4–18.0) was greater than that of entire animals in the study population (8.0 years; IQR 3.6–11.0; range 0.0–18.6). On average, entire females (6.4 years; IQR 1.0–10.2) died at a substantially younger age than neutered females (9.1 years IQR 6.2–11.9) (HR 0.74; 95% CI 0.54–1.00; *p* = 0.05), whilst the longevity of entire (8.3 years IQR 4.0–11.0) and neutered (9.0 IQR 5.5–10.9) males did not differ (*p* = 0.27).

Previous studies have suggested that coat colour may be a factor in longevity. Coat colours were evenly distributed within the deceased population, but there was no association between coat colour and longevity in these data (*p* = 0.22) (Table 2).

The age of death differed between groups of disorders (Table 3). The dogs in the group that was attributed to “old age”-associated diagnoses (14.0 years; IQR 12.4–15.0; range 10.0–18.6) and neurological disorders (12.5 years; IQR 6.7–14.5; range 0.1–17.0) had the longest median lifespan, whilst dogs with infectious diseases (0.4 years; IQR 0.3–0.6; range 0.0–7.8) and traumatic injuries (1.4 years; IQR 0.2–8.1; range 0.1–10.0) had the shortest median lifespans (Table 3). Bullmastiffs within the “old age” category could be considered as representing the maximum lifespan for the breed (HR 0.28; 95% CI 0.20–0.39; *p* < 0.001), while those with neurological disorders also lived significantly longer when compared to all other disorder groups (HR 0.55; 95% CI 0.36–0.85; *p* = 0.005). Dogs with behavioural disorders (HR 2.23; 95% CI 1.43–3.47; *p* < 0.001), infectious disease (HR 12.61; 95% CI 7.24–21.99; *p* < 0.001), traumatic injuries (HR 2.74; 95% CI 1.22–6.15; *p* = 0.01), intoxication (HR 3.62; 95% CI: 1.70–7.72; *p* < 0.001), alimentary tract disorders (HR 1.85; 95% CI 1.06–3.21; *p* = 0.03) and gastrointestinal disorders (HR 2.55; 95% CI 1.43–4.55; *p* = 0.001) had significantly shorter survival (Table 3).

### 3.5. Mortality

When considering the variation in lifespan, the common, underlying causes of death are particularly relevant to informing potential strategies for improving health and welfare. During the study period, 433 dogs died, and the main reason for death was categorised as either disorder/health condition (n = 271; 62.6%), unspecified (n = 65; 15.0%) or advanced age (n = 64; 14.8%) (Table 4). Significantly more of the deceased group were euthanised (n = 307; 70.9%) compared to those that were not (n = 35; 8.1%) (*p* < 0.001), with the euthanasia status of the remaining dogs not specified (n = 91; 21.0%). Of the animals that died during the period, there were 74 dogs (17.1%) with either no cause of death specified or for which a definitive disease diagnosis could not be made based on the recorded diagnostic investigations. At a fine-precision-level of diagnosis (n = 156), the most common causes of death attributed in the EPRs were “old age” (unspecified problems) (n = 44; 10.1%), osteoarthritis at multiple sites (n = 14; 3.2%), canine parvovirus (n = 13; 3.0%) and dog-related aggression (n = 9; 2.1%). When the causes of death were described at a grouped-precision level, the most common were mass lesions (n = 122; 28.2%), “old age” (n = 43; 9.9%) and musculoskeletal (n = 43; 9.9%), neurological (n = 23; 5.3%) and behavioural disorders (n = 21; 4.8%) (Table 3). There were 28 dogs in the population where a secondary disorder contributed to their death; the most common were musculoskeletal (n = 9; 32.1%), mass lesion (n = 6; 21.4%) and urogenital (n = 3; 10.7%). One dog had a third disorder associated with death, classified as musculoskeletal. The most common causes of death in dogs that were euthanised were mass lesions (34.5%) and musculoskeletal (12.7%), neurological (6.8%) and behavioural (6.8%) disorders. Dogs in the deceased group that were not euthanised were most commonly reported as having infectious diseases (11.4%), mass lesions (11.4%), gastrointestinal diseases (8.6%) or urogenital diseases (8.6%). The dogs with no specified euthanasia status died mostly of old age (23.1%), mass lesions (13.2%) and musculoskeletal disorders (4.4%), although a large proportion of these dogs did not have a specified cause of death (49.5%). The mortality of young dogs (<2 years old) was most frequently attributed to infectious disease (n = 13; 27.7%), whilst for adult dogs (≥2 years old), the most frequent cause of mortality was mass lesions (n = 122; 31.6%). Table 5 lists the most frequent disorders at death within the three most common grouped-precision-level causes of death. Lymphoma (n = 21; 17.2%; 95% CI 11.5–24.9) was the most reported mass lesion associated with death, whilst osteoarthritis at multiple sites (n = 14; 32.6%; 95% CI 20.5–47.5) was the most frequent musculoskeletal disorder.

Age, neuter status and weight were associated with the cause of death, whilst colour and sex were not (Table 6). Multivariate regression analysis revealed that weight had a small but statistically significant impact on mass-lesion-related deaths (OR 1.02; 95% CI 1.01–1.04; *p* = 0.007). Age (OR 1.01; 95% CI 1.00–1.01; *p* = 0.054) and, more substantially, neuter status (OR 2.09; 95% CI 1.04–4.46; *p* = 0.036) had a statistically significant effect on mortality from musculoskeletal disorders. Gonadectomy status also affected urogenital-related deaths with neutering markedly reducing mortality from urogenital disorders in the study population (OR: 0.14, CI: 0.02–0.52; *p* = 0.003). The top three fine-level urogenital diagnoses were pyometra (41.7%), chronic kidney disease (33.3%) and acute kidney failure (16.7%).

## 4. Discussion

The current study presents an analysis of the demography, mortality and longevity of Bullmastiffs attending a large sample of Australian veterinary practices. The Bullmastiff was found to have an average lifespan of 8.5 years, with mortality most frequently attributed to mass lesions, old age and musculoskeletal, neurological and behavioural disorders. The shortened longevity of the breed relative to published data from the broader population of dogs aligns with previous reports from UK owner surveys [5,7]. This study provides further evidence for a range of disorders found in larger dogs contributing to mortality, particularly mass lesions and musculoskeletal disorders [9,14,24,25,26]. However, there was little evidence of brachycephalic problems causing death in this breed, with very low numbers of ophthalmologic (n = 2) and respiratory disorders (n = 4) (Table 3). Female Bullmastiffs had shorter lifespans than neutered females, entire males and neutered males. Gonadectomy significantly reduced female mortality from urogenital causes, which has not been presented previously in this breed. Together, these findings contribute to documenting the health and disease status of Bullmastiffs in Australia and may provide useful information for improving welfare, breed management and the Bullmastiff quality of life.

The number of ANKC-registered Bullmastiffs declined over the study period, with the number of registrations in 2017 being 38.4% less than in 2008. The birth rates as a percent of dogs of this breed visiting veterinary practices in each year also comparably declined, accounting for only 17.2% of patients in 2017, falling from 45.4% in 2011. VetCompass UK studies revealed similar declining registration trends in the two most popular large dog breeds; the Labrador (9.6% in 2004 to 5.8% in 2013) and German Shepherd (3.5% in 2005 to 2.2% in 2013) [18,27]. In contrast, smaller brachycephalic breeds such as the French Bulldog and Bulldog have been popularised in the UK and America [28]. Additionally, ANKC registration data revealed that shorter and smaller breeds with a high cephalic index (brachycephalic head shapes) are on the rise in Australia [29]. A UK owner survey found that factors such as appearance and companionship are increasingly important considerations for owners who select small brachycephalic breeds [30]. The American Kennel Club similarly described the functionality (health, trainability) of breeds as a less important trait in owner selection [31]. Recent trends in dog ownership favour small indoor companions over previously popular large mesaticephalic breeds, which were once desired for protection and hunting but require more outdoor space and regular exercise [32]. The decrease in ANKC Bullmastiff registrations as well as the decreasing birth rates reflect the current international preference towards smaller dog breeds.

The current study found a predominance of young Bullmastiff dogs attending Australian clinics, with 51.4% of records for patient less than three years old (Table 1) and a median study population age of 2.8 years. This was similar to Bulldogs attending clinics in the UK [3]. The young median age may be attributed to wellness visits at the start of a dog’s life for vaccinations and desexing. In 2017, 54.1% of Bullmastiffs attending veterinary practices in Australia were under four years old and were most commonly presented for vaccinations (57.7%). To account for the age profile of the study population, young dogs were excluded for analyses of mortality but were included for growth rate analyses. An Italian study reported similar findings, with two peaks in veterinary visits for dogs; at age 1–2 and 10 years [33].

As expected, rapid growth occurred in the first year of life for the dogs in this study, whilst growth in the second year slowed and plateaued in adult years followed by a decline from 8.3 to 12.5 years of age (100–150 months). The presented body weight curves show a similar profile to those of other large breeds, such as German shepherds and Rottweilers in the UK, both showing weight declines from 12 years of age [27,34]. English Mastiffs in the UK achieved 99% of their adult body weight in the first 65.2 weeks of age [35], similar to the Bullmastiffs in the present study. The significant decrease in weight (17.0% in females and 24.3% in males) from 12.5 years of age has implications for the Bullmastiff population. The change in body weight likely reflects the loss of muscle condition and reduced adipose tissue in dogs due to health conditions that reduce physical activity. This information may provide a basis for veterinarians to monitor and advise on subclinical metabolic and joint diseases and recommend optimal geriatric diets with higher nutrient density to maintain a healthy body weight.

The mean body weights of the adult dogs were less than the ANKC standard weight range, with 40.9% of female records and 44.9% of male records below the range. Patients described by veterinarians as underweight (n = 66) or patients that had conditions that contribute to significant weight loss were excluded from the analysis of body weight in this study; hence, the percentage of dogs weighing less than the breed standard is difficult to explain, as breed identification was checked by veterinarians. Breed identification following examination by a veterinarian is the best available information on breed in this study, but we cannot rule out the misidentification of some dogs with a lack of information on origin [29], a limitation acknowledged in other studies of veterinary clinical records. We note that the breed standard does state that Bullmastiffs may not conform to the weight range, and the standard is applied to dogs that have been optimized for show conditions and not applied to others. The issue of breed identification is common to studies based on primary care veterinary data or where dogs are included without confirmation of registration status. Body weight was not routinely recorded in EPRs, which may reflect the difficulty of positioning a large, heavy dog on a weighing scale, with only 25.3% of adult dogs in this study having a body weight recorded in the database. Clinical records in the UK also showed a relatively low percentage of weight records for adult dogs, with only 50.3% of the total population [6]. These data are a cause for concern, as body weight declines in older dogs are substantial and perhaps insufficiently monitored in the breed, which may impact negatively upon nutritional advice and drug dose rates.

The reported 8.5-year median longevity of the Bullmastiff is similar to previous reports in UK health and British questionnaire studies, with Bullmastiffs having shorter lifespans than the broader dog population, reported in those studies to be 11.4 years and 12 years, respectively [5,7]. This is consistent with other studies that have described a shorter lifespan for large dog breeds. The relationship between size and lifespan is not clear and is complicated by the tendency for large dog breeds to be more inbred [2]. The estimated inbreeding co-efficient for Australian Bullmastiffs is 0.039, based on molecular SNP data analysis [36], which may have an effect. The expression of genes associated with the skeletal size and body mass of large dog breeds appears to predispose large dogs to growth-related musculoskeletal disorders, osteosarcomas and shortened lifespans [36]. The present study found mortality due to musculoskeletal disorders (9.9%) that is similar to data from Bullmastiffs attending USA veterinary teaching hospitals. In the USA study, elevated levels of musculoskeletal disorders, specifically osteoarthritis and ruptured cranial cruciate ligament, were documented and align with EPR studies that demonstrated that giant breeds commonly die or are euthanised for musculoskeletal disorders and gastrointestinal causes, with hip dysplasia being a common cause of mortality for giant dogs in a Danish kennel club study [9,24]. A UK study found osteoarthritis to be the most severe condition impacting dog welfare [37], and evidence showed that a decline in or loss of mobility created greater problems for the home management of giant breeds compared to other breeds. Accordingly, early treatment which is effective, maintains muscle mass and manages pain and surgical correction when possible is necessary, especially for large dog breeds which are predisposed to dying early of these disorders.

The current study revealed that the median lifespan of neutered females (9.1 years) was 2.7 years longer than that of entire females (6.4 years), whilst the gonadectomy status in males had no effect. Previous studies concur with this difference: a UK questionnaire study on kennel club dogs reported that neutered females (12.0 years) lived longer than entire females (10.1 years), and EPR data revealed that neutered Bulldogs (8.0 years) outlived entire animals (6.7 years) [3,7]. The analysis of two large EPR datasets from Vetcompass UK and the Veterinary Medical Databases (VMDBs) in the USA associated neutering with greater longevity for half of the breeds reported, which was especially true for neutered females [38]. The current study revealed that neutering decreased the risk (OR: 0.14) of death by a urogenital disorder, specifically pyometra which accounted for 41.7% of urogenital-related deaths. The current study did not find any deaths attributed to dystocia, suggesting that the Bullmastiff breed does not share the increased risk associated with brachycephalic breeds. It is also worth noting that the date of neutering for the dogs in the current study was not available in the EPRs, so the implications of the long-term presence or absence of sex hormones on the longevity and mortality of Bullmastiffs remains unknown.

This study reported that 70.9% of dogs were euthanised, whilst 20.1% of dogs did not have a reported mode of death; hence, it is possible that the euthanasia rates could be underestimated. Owned and kennel-club-registered dogs in the UK had higher euthanasia rates: 86.4% and 79.58%, respectively [6,26]. Similar trends to those in the current study were evident in the USA, where a veterinarian surveillance study reported a euthanasia rate of 71%, and 68.5% of purebred dog deaths were assisted in referral practices [25,39]. Higher euthanasia rates reflect the increased prioritisation of quality of life and welfare in companion animals, based on professional guidelines such as the American Veterinary Medical Association (AVMA)’s regularly updated euthanasia guidelines to ensure that assisted death is conducted appropriately [6]. Furthermore, quality of life has been reported as an important factor in euthanasia decisions by British owners [40]. Recent research is reflective of efforts to improve the health and welfare of dogs through managing controllable risk factors (e.g., weight and neuter status) for disease/lifespan as well as optimising the use of quality-of-life measures in monitoring end-of-life care [1,16,41].

The most frequent cause of mortality in the current study was mass lesions (28.2%). EPR data in the UK concur with these findings, with the most frequent causes of death in owned dogs being neoplastic diseases (16.5%), followed by musculoskeletal disorders (11.3%) and neurological disorders (11.2%) [6]. An equivalent study of purebred and mixed-breed dogs in Denmark found old age (20.8%), cancer (14.5%) and behavioural problems (6.4%) to be the main causes of death [9]. A kennel club study of dogs in the UK reported that the main causes of death were old age (13.8%), unspecified cancer (8.7%) and, in contrast to the current study, heart failure (4.89%) [26]. It is evident that cancer is a prevalent cause of death in many dog populations. Certain breeds, notably larger dog breeds such as the Irish Wolfhound (22%), Bernese Mountain Dogs (34.2%) [8,42] and the Dogue de Bordeaux (32.4%) [12], are at greatest risk, which is consistent with a genetic contribution to susceptibility in dogs [43,44]. It may also be argued that increased disease surveillance in dogs due to growing concern for their health and welfare may further contribute to the increasing number of cancer diagnoses [45].

The high prevalence of mass lesions in the current study was consistent with the increased risk of lymphoma reported previously in English and Australian Bullmastiff populations [13,14,46,47]. It was also evident that many mass lesions presented to veterinary practices were not investigated in detail before euthanasia or death occurred; thus, an underrepresentation of specific neoplastic disorders such as lymphomas and osteosarcomas is likely. Lymphoma in Bullmastiffs has the highest incidence in some sub-populations or lines, and although in a relatively small population of dogs there is an increased level of inbreeding, the genetic basis of lifespan and lymphoma susceptibility is complex [45,48]. However, in other studies, the level of inbreeding has been shown to have an impact on individual lifespan in Golden Retrievers, without specifying the causes of death [2]. The median age of dogs dying from mass lesions in the current study was 9.4 years (IQR 6.6–10.8), which is greater than the peak period for breeding, suggesting there may be little impact on heritability [49]. However, in an analysis of data previously collected on 121 reported deaths in a sub-population of Bullmastiffs with an increased incidence of lymphoma, the median age of death was 6.1 (IQR 5–7.75). Interestingly, females in this group lived on average longer than males (*p* < 0.05) (Supplementary Figure S1). Compared with the EPR data, this suggests that lymphoma has an impact on individual lifespan and would tend to reduce the median longevity in the breed.

The age at death in this study illustrates bimodal peaks in the first year of life and at 8–10 years. This warrants further investigation into the different causes of mortality for differing age groups. The most common cause of death in the dogs dying at less than one year of age (9.2%) was infectious diseases (30.0%), mainly parvovirus (n = 11). Recent findings in Australia show that variation in vaccination recommendations by veterinarians, as well as the under-recognition of infection risks in different regions, contributes to parvovirus mortality [50]. Hazard ratios revealed significantly shorter lifespans because of gastrointestinal disorders, traumatic injuries and behavioural issues related to aggression. UK data revealed similar trends with the mortality of dogs less than three years old related to behavioural, gastrointestinal and traumatic conditions [6]. An analysis of Australian dogs did not find aggressive behaviour in Bullmastiffs, and previous reports of euthanasia as a result of aggression are not apparent [51]. Conversely, in the current study, 4.8% of dogs were euthanised as a result of aggressive behaviour towards other dogs, which is similar to rates reported in a UK study of the Bulldog, with 5.5% euthanised due to undesirable behaviour [3].

Dogs reported as dying of old age (9.9%) by definition had the greatest longevity, but those classified with neurological disorders (5.3%) also had longer lifespans (median longevity 14.0 years and 12.5 years, respectively) when compared to the other disorder groups. The literature has not reported in depth on neurological disorders as a cause of mortality in giant breeds. The EPR exploration of owned dogs in the UK avoided the use of “old age” as a cause of death because it does not describe the pathological processes leading to death [6]. Dogs dying of old age in the current study had nonspecific presenting complaints not related to disorders of one body system (e.g., lethargy, falling, depressed) and no clinical record of underlying conditions contributing to death. This is similar to the methodology of UK health and kennel club survey studies as well as a Danish kennel club study [7,26,42]. The current study chose this methodology to avoid an excess of dogs given no diagnosis at death with data completeness for mortality being only 82.9%.

The current study has limitations. The total number of males was marginally, but significantly, greater than that of females. This has been noted for dogs in other studies, e.g., [10], but there is no known explanation for why this is observed. Electronic patient records were not created for research purposes; rather, they were produced solely for patient care and thus were not subject to bias introduced by recruitment to prospective studies. Nonetheless, the data were limited by incomplete records and records with no examination text or examination text which was not descriptive enough to assign an appropriate cause of death. These factors resulted in 16.2% of dogs not being given a diagnosis, which was similar in an EPR study in the UK, which reported 13.0% of dogs without a diagnosis at death [6]. Furthermore, when owners and veterinarians opted for euthanasia, there was no further investigation or clinical recording. For example, clinical notes prior to death state presenting signs such as “lethargic” when this is not a disease diagnosis but rather contributes to the underrepresentation of certain mortality causes. As well as this, the neuter status “entire” was the default setting for the dogs, so some neutered dogs may not have been recorded as castrated or spayed. This study was limited by the inclusion of only VetCompass Australia participating practice attending dogs, which were located only in the eastern states of Australia (NSW, QLD, VIC) with no Bullmastiffs recorded from practices in other states. The patient data from this study were also not representative of dogs which had not received veterinary care immediately prior to death or had died at home.

## 5. Conclusions

This study of 2771 dogs, including 433 deceased dogs, provides a detailed analysis of the demography, longevity and mortality of Bullmastiff dogs in Australia. The breed has a shortened lifespan relative to the broader population of dogs and died from similar conditions to breeds of dogs reported in the UK, however with a higher rate for cancer, which is consistent with findings in other giant breeds. The analysis also revealed that neutering has a significant impact on lifespan and is protective against urogenital mortality, which provides useful practical information for the Bullmastiff breed. The results of this study provide evidence that may form the basis for the management of health in the breed and highlight those conditions which should be prioritised to improve the overall welfare of Bullmastiff dogs. The value of EPR exploration is a key element of this study, supporting the usefulness of this epidemiological methodology in companion animal health.

## Figures and Tables

**Table 1:** Demography of Bullmastiffs under veterinary care at practices participating in the VetCompass Australia programme from 1 January 2008 to 31 December 2017.

Variable	Category	Count in Population	Frequency Population (%)	Count (Deceased Dogs)	Frequency in Deceased Dogs (%)	Frequency of Deceased in Total Population (%)
Sex	Female	1259	45.4	187	43.2	14.9
	Male	1491	53.8	243	56.1	16.3
	Unknown	21	0.8	3	0.7	14.3
Neuter status (female)	Neutered	609	48.3	127	67.9	20.9
	Entire	650	51.6	60	32.1	9.2
Neuter status (male)	Neutered	576	38.6	124	51.0	21.5
	Entire	915	61.4	119	49.0	13.0
Neuter status (unknown)	Unknown	21	100.0	3	100.00	14.3
Colour	Brindle	442	16.0	78	18.0	17.6
	Fawn	561	20.2	80	18.5	14.3
	Red	1254	45.2	205	47.3	16.3
	Other	302	10.9	34	7.9	11.3
	Not described	212	7.7	36	8.3	17.0
State	NSW	1951	70.4	307	70.9	15.7
	QLD	328	11.8	39	9.0	11.9
	VIC	492	17.8	87	20.1	17.7
Female adult body weight (aged ≥24 months) (kg) ^a,b^	<25.0	34	4.4	16	12.6	
	25.0 to 29.0	94	12.1	10	7.9	
	30.0 to 34.0	132	17.0	18	14.2	
	35.0 to 39.0	123	15.8	12	9.4	
	40.0 to 44.0	121	15.6	22	17.3	
	45.0 to 49.0	110	14.2	18	14.2	
	50.0 to 54.0	89	11.5	18	14.2	
	55.0 to 59.0	34	4.4	5	3.9	
	60.0 to 64.0	26	3.3	5	3.9	
	≥65	14	1.8	3	2.4	
Male adult body weight (aged ≥24 months) (kg) ^a,b^	<25.0	7	0.8	12	7.2	
	25.0 to 29.0	82	9.0	14	8.4	
	30.0 to 34.0	105	11.5	12	7.2	
	35.0 to 39.0	98	10.8	18	10.8	
	40.0 to 44.0	117	12.8	27	16.3	
	45.0 to 49.0	124	13.6	23	13.9	
	50.0 to 54.0	96	10.5	20	12.0	
	55.0 to 59.0	136	14.9	15	9.0	
	60.0 to 64.0	83	9.1	11	6.6	
	≥65	63	6.9	14	8.4	
Age ^c^	<3.0	13,010	51.4	63	14.5	
	3.0 to <6.0	5450	21.5	65	15.0	
	6.0 to 9.0	3781	14.9	105	24.2	
	9.0 to <12.0	2296	9.1	110	25.4	
	12.0 to <15.0	651	2.6	65	15.0	
	≥15.0	129	0.5	25	5.8	

^a^ Count of female and male body weights included all records from body weight data. ^b^ The weights of the Bullmastiffs in the deceased population were the last recorded weight of the dog. ^c^ Counts were of records not individual dogs for whole population.

**Table 2:** The longevity and survival analysis of deceased Bullmastiffs in records from the VetCompass Australia programme from 1 January 2008 to 31 December 2017 (n = 433).

Variable	Category	Survival (Years)		P (log Rank)	Hazard Ratio ^a^	95% CI ^a^
		Median	IQR			
All dogs	All	8.5	5.3–11.2			
	Male	8.5	5.2–11.0	0.38		
	Female	8.3	5.4–11.5			
Weight ^b^	Overweight	8.7	7.5–9.5	0.98		
	Not overweight	9.0	6.0–11.1			
Coat colour	Brindle	8.3	4.3–10.9	0.22		
	Fawn	8.1	5.5–10.3			
	Red	9.1	6.0–11.9			
	Not described	7.7	3.1–11.2			
	Other	6.2	3.1–12.8			
Dogs with known gonadectomy status	All	8.5	5.3–11.2	0.18		
	Male entire (n = 118)	8.3	4.0–11.0	0.27		
	Male neutered (n = 125)	9.0	5.5–10.9			
	Female entire (n = 58)	6.4	1.0–10.2	0.05	0.74	0.54–1.00
	Female neutered (n = 129)	9.1	6.2–11.9			

^a^ Only significant findings of survival analysis are reported. ^b^ Weights of deceased dogs were classified as overweight or not overweight compared to the ANKC breed standard. Adults 10% over the breed standard weight range were classified as overweight.

**Table 3:** Survival associated with causes of death of Bullmastiffs in this study (n = 433).

Disorder Group	Count	Prevalence (%)	Survival (Years)		
			Median	IQR	*p* Value ^a^
Mass lesion	122	28.2	9.4	6.6–10.8	0.7
Old age	43	9.9	14.0	12.4–15.0	<0.001
Musculoskeletal	43	9.9	9.1	6.5–12.3	0.3
Neurological	23	5.3	12.5	6.7–14.5	0.005
Behavioural	21	4.8	4.0	2.4–7.0	<0.001
Infectious	15	3.5	0.4	0.3–0.6	<0.001
Alimentary tract	13	3.0	6.0	0.4–8.0	0.03
Cardiac	12	2.8	8.0	5.9–12.6	0.6
Gastrointestinal	12	2.8	2.8	1.3–8.9	<0.001
Urogenital	12	2.8	7.8	5.4–10.6	0.8
Nutritional	8	1.8	8.7	6.9–10.0	0.6
Intoxication	7	1.6	4.8	1.8–6.3	<0.001
Traumatic injury	6	1.4	1.4	0.2–8.1	0.01
Inflammatory	4	0.9	10.3	9.0–10.9	
Integumentary	4	0.9	7.0	3.2–10.8	
Respiratory	4	0.9	8.2	5.8–9.3	
Endocrine/metabolic	3	0.7	10.4	7.1–10.4	
Other conditions	3	0.7	9.3	8.8–9.9	
Haematopoietic	2	0.5	4.4	2.6–6.2	
Ophthalmologic	2	0.5	3.5	1.8–3.6	

^a^ Survival analysis was not performed on disorder groups with counts less than 5. *p* values relate to groups which lived significantly longer or shorter than all other disorder groups.

**Table 4:** The euthanasia status and main categories for causes of death of Bullmastiffs in the VetCompass Australia programme from January 2008 to December 2017 (n = 433).

Cause of Death		Count	Frequency (%)	95% CI
Euthanasia status	Euthanised	307	70.9	66.5–75.0
	Not specified	91	21.0	17.4–25.1
	Died	35	8.1	5.9–11.0
Disorder, health condition		271	62.6	58.0–67.1
Unspecified		65	15.0	11.6–18.4
Advanced age		64	14.8	11.4–18.1
Problematic behaviour		21	4.8	2.8–6.9
Accident, traumatic injury		12	2.8	1.2–4.3

**Table 5:** The most prevalent causes of mortality in the Bullmastiffs in the VetCompass Australia programme from January 2008 to December 2017. The most frequent specific diagnosis of death for each disorder group is described, and the frequency of these within the disorder groups is described (n = 110).

Disorder Group	Specific Diagnosis	Count	Frequency of Specific Diagnosis (%)
Mass lesion	Lymphoma	21	17.2
	Mass lesions	6	4.9
	Neoplasms	10	8.2
Musculoskeletal	Osteoarthritis, multiple sites	14	32.6
	Degenerative joint disease	7	16.3
	Arthritis (septic and non-septic)	9	20.9
Old age	Old age (unspecified problems)	43	100.0

**Table 6:** Results of univariate and multivariate regression models for grouped-precision-level causes of death. When one or more signalment factors showed significance in the univariate regression model, they were then analysed in the multivariate regression model.

Disorder Group	Variable	*p* Value (of Univariate Regression)	*p* Value (of Multivariate Regression)
Mass lesions	Age	0.0133	0.063
	Neutered	0.005	0.082
	Weight	0.003	0.007 (OR: 1.02; CI: 1.01–1.04)
Musculoskeletal	Age	0.045	0.054 (OR: 1.01; CI: 1.00–1.01)
	Neutered	0.021	0.036 (OR: 2.09; CI: 1.04–4.46)
Old age	Age	<0.000 (OR: 1.05; CI: 1.04–1.07)	
Neurological	Age	0.0010 (OR: 1.01; CI: 1.00–1.02)	
Behavioural	Age	0.001 (OR: 0.99; CI: 0.97–0.99)	
Infectious	Age	0.000	0.001 (OR: 0.97; CI: 0.92–1.00)
	Neutered	0.001	0.434
	Weight	0.015	0.454
Alimentary	Age	0.020	0.987
Gastrointestinal	Age	0.003	0.010 (OR: 0.99; CI: 0.97–1.00)
	Neutered	0.006	0.022
Urogenital	Neutered	0.003 (OR: 0.14; CI: 0.02–0.52)	
Intoxication	Age	0.014 (OR: 0.98; CI: 0.96–1.00)	
Traumatic injury	Age	0.013	0.083
	Weight	0.022	0.077

## Data Availability

The datasets provided and analysed during the current study are not publicly available records; the data are part of a larger collaboration with VetCompass Australia, with research still ongoing. Data are available from the corresponding author on reasonable request.

## Supplementary Materials

The following supporting information can be downloaded at https://www.mdpi.com/article/10.3390/ani14233419/s1, Figure S1: Age at death for males and females in a sub-population of Bullmastiff dogs.

## Abbreviations

EPR: electronic patient record; OR: odds ratio; HR: hazard ratio; IQR: interquartile range; CIs: confidence intervals; ANKC: Australian National Kennel Club; VMDBs: Veterinary Medical Databases; AVMA: American Veterinary Medical Association.
