# Supplementary material for: The Demography, Longevity and Mortality of Bullmastiffs Attending Veterinary Practices in Australia
Source: Animals (Basel). 2024 Nov 26;14(23):3419. doi: 10.3390/ani14233419 (PMC11640064; doi:10.3390/ani14233419)

**Supplementary Figure S1**. Age at death for males and female in a sub-population of Bullmastiff dogs (n=121). Box plots showing interquartile ranges.

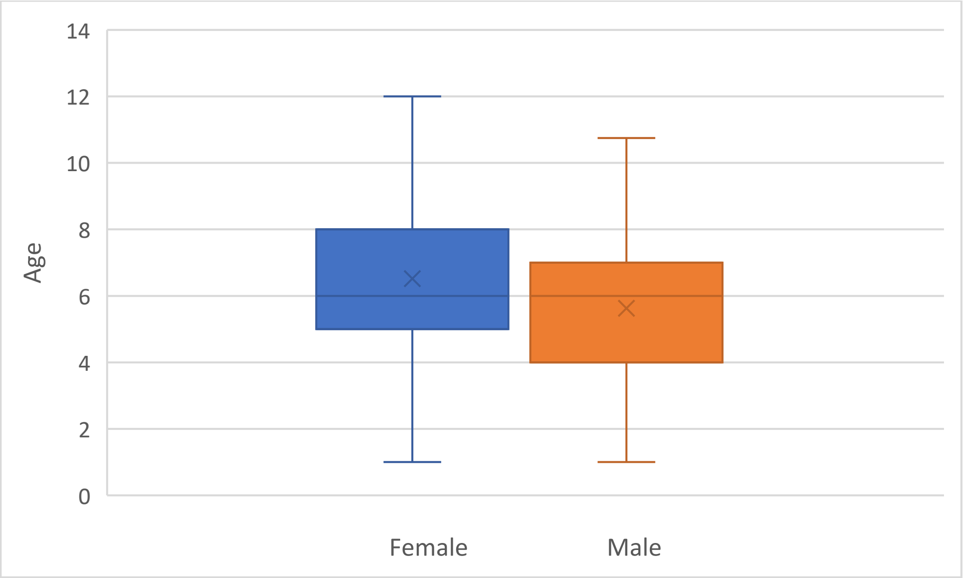

Supplement: Supplementary file 1 [file animals-14-03419-s001.zip › animals-3222713-supplementary.pdf]
